# PRESIDENTIAL SYMPOSIUM: EMBRACING OPPORTUNITIES AND CONFRONTING CHALLENGES IN GLOBAL AGING RESEARCH

**DOI:** 10.1093/geroni/igae098.1060

**Published:** 2024-12-31

**Authors:** Jennifer Ailshire

**Affiliations:** Chair

## Abstract

This BSS Presidential Symposium highlights the promise and potential of global studies of older adults to bring new insights to our understanding of the aging process in an ever changing and increasingly diverse world. The Symposium brings together scholars from Gerontology, Sociology, and Economics to highlight the multidisciplinary perspectives on aging in a global context. Jinkook Lee will provide an overview of what we can learn from cross-national comparisons of aging populations and how we can use evolving data resources to address the challenges of aging populations around the world. Three empirical presentations follow from Yaun Zhang, Mateo Farina, and Nekehia Quashie, with different perspectives on how to use global aging surveys to advance our understanding of the social determinants of cognitive health across diverse country contexts. These presentations, which focus on education, early life conditions, and family relationships span countries from Latin America, Asia, Europe, and the United Kingdom as well as the United States. The session also highlights the value of using harmonized studies of aging across different countries to demonstrate how inequality differs across contexts and identify the drivers of disparities in cognitive health and dementia risk. By integrating diverse disciplinary perspectives and utilizing harmonized studies, this Symposium will demonstrate how researchers can use cross-national comparative research to examine questions that can inform efforts to enhance the well-being of older adults worldwide and address the multifaceted challenges posed by global aging.

